# A proposal for capturing interaction and effect modification using DAGs

**DOI:** 10.1093/ije/dyac126

**Published:** 2022-06-13

**Authors:** John Attia, Elizabeth Holliday, Christopher Oldmeadow

**Affiliations:** School of Medicine and Public Health, The University of Newcastle, NSW, Australia; Division of Medicine, Hunter New England Health Local Health District (HNELHD), NSW, Australia; Hunter Medical Research Institute, NSW, Australia; School of Medicine and Public Health, The University of Newcastle, NSW, Australia; Hunter Medical Research Institute, NSW, Australia

## Background

Directed acyclic graphs (DAGs) are a helpful tool for depicting causal relationships among variables and are widely used to understand the impact on causal effect estimates when different variables are conditioned upon. Despite their significant utility, a relative gap remains in the inability to graphically represent interaction or effect modification in the conventional DAG framework.

In 2007, VanderWeele and Robins published two classic papers on interaction and effect modification based on DAG theory. The first^1^ proposed a system for classifying an effect modifier variable into one of four different types, according to its causal relationships with variables constituting the cause and the effect: direct effect modification, indirect effect modification, effect modification by proxy and effect modification by a common cause. Although this classification captured different causation structures, it did not address graphical representation of effect modification. The second paper^2^ showed how Rothman’s sufficient component cause model could be incorporated into the causal DAG framework, allowing the definition of independencies arising from conditioning on a common effect. However, this framework could only be applied to binary exposures and outcomes. For those not well versed in causal inference literature, these papers were also mathematically dense and conceptually challenging.

Although the terms ‘interaction’ and ‘effect modification’ are frequently used interchangeably, they have different meanings in causal inference, as elaborated by VanderWeele.^3^ Limiting cases were also described in which interaction can happen without effect modification and effect modification can happen without interaction.^3^

In a commentary,^4^ Weinberg lamented that the conceptualizations offered by VanderWeele and Robins were ‘DAG-specific’ and not necessarily intended to be ‘biologically meaningful’. Weinberg spoke for many in the epidemiologic community when she expressed her frustration that ‘many important kinds of causal relationships are not captured graphically by DAGs’, such as effect modification. Weinberg suggested a number of ways in which DAGs could incorporate ‘arrow on arrow’ paths to capture interaction and effect modification. However, although conceptually appealing and heuristically helpful, these relationships cannot be ‘read’ as conventional DAGs and cannot be drawn using standard DAG software (e.g. DAGitty^5^).

Nilsson *et al*.^6^ recently proposed a new type of DAG for capturing interaction—the interaction DAG (IDAG), which replaces the outcome node with a node representing the causal effect. Although this notation preserves the usual DAG conventions regarding reading and interpreting the IDAG, this framework separates the interaction from the main effect, i.e. requires two separate DAGs to capture main effects and interactions. In some situations it may be useful to show both main effects and interactions in a single DAG, and in this paper we propose a simple approach to graphically depict this in a manner conforming to usual DAG conventions and which can be drawn using available DAG software.

## Interaction

Interaction occurs when there is interest in the causal effects of two exposures on an outcome and the effect of either exposure depends upon the value of the other, i.e. the causal effect of each exposure ‘varies’ across levels of the other. In the presence of interaction, the expected value of the outcome cannot be derived from a simple arithmetic function (e.g. sum or product) of the two independent exposure effects for all members of a population. To define what is ‘expected’, either an additive or multiplicative model may be used, usually when estimating absolute and relative measures of effect, respectively. However, additive models can also be applied to relative measures of effect and it is recognized that interaction is always judged with respect to the effect measure or scale chosen and the model used to define what is ‘expected’, given the two separate exposures.

**Figure 1:**
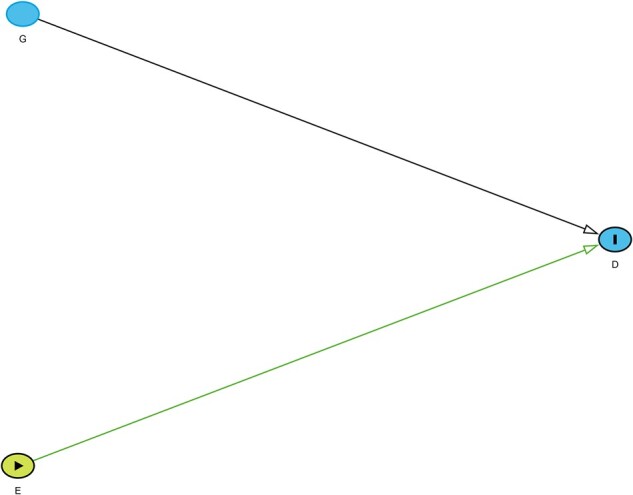
DAG adapted from VanderWeele (2009).^3^*E* is a weight-loss medication, *G* is exercise and *D* is child’s weight. There is nothing in this DAG suggesting the possibility of interaction between the effects of *E* and *G* on *D*.

In his 2009 paper, VanderWeele^3^ describes an example in which a binary exposure E is a randomized weight-loss drug for obese children, a binary exposure G is exercise and the outcome D is a child’s weight after a trial (Figure 1). An interaction (on the additive scale) is postulated between exercise and drug therapy, such that:

$$E\left(D|E=1,G=1\right)-E\left(D|E=0,G=1\right)\neq E\left(D|E=1,G=0\right)-E\left(D|E=0,G=0\right)$$

That is, the effect of the weight-loss drug upon final weight differs according to whether a child has exercised. However, although the DAG indicates that E and G are both causes of D, there is nothing to indicate the proposed interaction between the two causes.

Although there is nothing wrong with omitting the interaction term in terms of what arrows in causal DAGs represent (causal effects in at least one person of the population), we believe that explicitly capturing interaction is likely to be helpful for a few reasons:

- As a heuristic in helping researchers explicitly articulate their hypotheses about cause and effect. As Miguel Hernán has said: ‘Draw your assumptions before drawing your conclusions.’^7^ We argue (as does Clarice Weinberg in the quoted editorial) that as a tool for communication, capturing interaction and effect modification explicitly in a DAG is a step forward.
- Confounding bias: a DAG that explicitly captures interactions would potentially prompt the researcher to think about factors that increase the likelihood of having both exposures present simultaneously; if such factors also affect the risk of the outcome, then these would constitute a back-door path. Although such back-door paths could also be captured by having arrows into both exposures, the notation in Figure 1 does nothing to prompt such thinking.
- Faithfulness: this is the assumption that arrows indicate a probabilistic dependence of the child node on its parent. When there is qualitative interaction (effect of an exposure on outcome occurs in the opposite direction within levels of a third variable) the average causal effect can cancel out giving the appearance of d-separation between the exposure and outcome, when in reality there is a causal effect in at least one member of the population. Allowing interactions to be included in the DAG might prompt the researcher to think about this possibility, although we acknowledge that this coincidental and exact cancellation of effects is likely to be a rare event.

**Figure 2:**
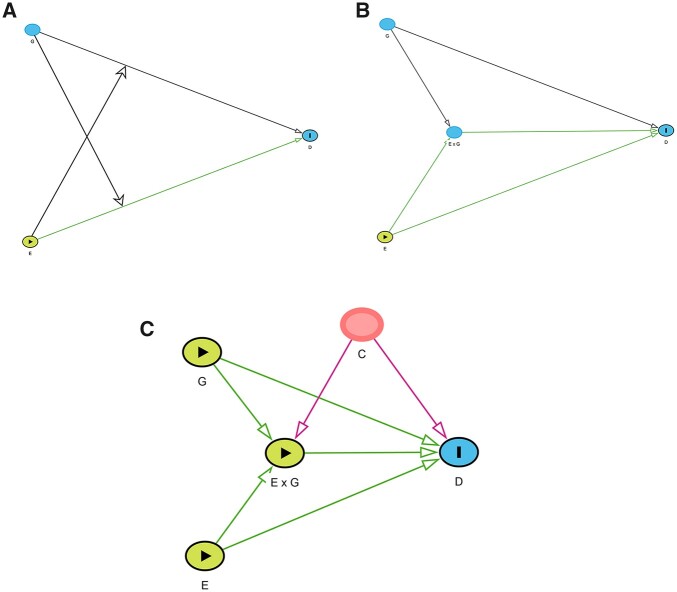
(a) Proposal by Weinberg (2007) for capturing interaction from Figure 1. (b) DAG representing the independent effects of exposures *E* and *G* and their combined effect E × G (interaction effect) on *D*. (c) Potential confounding affecting an interaction effect.

To graphically represent interaction between two exposure effects, Weinberg^4^ suggested the addition of arrows emanating from each exposure (see Figure 2a). However, whilst conceptually appealing, this representation can neither be interpreted using conventional DAG theory nor depicted using available DAG software. This limits its utility with respect to defining causal effects.

We suggest that interaction between the effects of E and G upon D could be graphically represented via the DAG shown in Figure 2b.

Here, inclusion of the E × G node represents an additional effect on *D* that is present only when *E* and *G* are both present; it formally represents the assumption that the causal effect of exposure E on outcome D (on the scale of interest) depends upon the value of G, or equivalently that the causal effect of exposure G on outcome D depends upon the value of E. A biological example of interaction is seen with smoking, asbestos and lung cancer; although both smoking and exposure to asbestos increase the risk of lung cancer, in the presence of both factors together there is an additional risk of the outcome, which can be represented by the interaction *E* × *G* node.

The graphical representation in Figure 2b has previously been suggested by both Slaug *et al*.^8^ and Spiller *et al*.^9^ In both cases, this graphical depiction has been presented as an intuitive way to express interaction and used to guide other analyses without further exploration of its properties in the conventional DAG framework. We describe key features of this graphical representation of interaction below:

- Because interest is in the causal effect of exposures E and G, assuming interaction between E and G, causal diagrams can be constructed by specifying all three nodes E, G and E × G as exposure variables. This implies interest in the causal effects of E, G and E × G, allowing for biasing (e.g. confounding) paths between any exposure and the outcome by other, causally related variables, which can be depicted in the usual way.
- The DAG depicts the assumption that each exposure E and G has a direct causal effect on D, but also represents in an intuitive way that when *E* and *G* are present together there is an additional effect on *D*. It should be noted that with the inclusion of the interaction node, these ‘main’ effects no longer represent average causal effects in the population, but rather the average causal effect of each exposure at a specified (e.g. reference) value of the other exposure. One assumption of DAGs is that arrows denote probabilistic relationships, whereas the arrows from *E* and *G* into *E* × *G* might appear deterministic, i.e. where *E* and *G* are present, *E* × *G* is always present. We argue that these arrows are not in fact deterministic and do not violate DAG conventions. The presence of *E* does not influence the presence of *G* and vice versa, hence the arrow from *E* to *E* × *G* still encodes a probabilistic dependence. Likewise, not everyone with *E* and *G* together will develop *D*, i.e. there will be some people with *E* and *G* who do not develop *D*, so this arrow also encodes a probabilistic dependence. We grant that this probability may be high, but as long as this probability does not equal 100%, the assumptions behind DAGs are not violated. On the other hand, if this probability equals 0, then we may be led to close some back-door paths that are not present and, at worst, this may lead to a small degree of overadjustment.
- The interaction DAG depicts the assumed causal effect of E × G, representing an average causal effect for one or more patterns of joint values of *G* and *E*. These potential patterns are defined according to the scales of *G* and *E* and the corresponding definitions of ‘main’ effects. This node is thus a probabilistic common effect of *E* and *G*, conditioning on which induces conditional association between *E* and *G* in at least one of the strata of *D*.^9^ For the case of binary *E* and *G*, the arrow between *E* and *D* in this DAG would imply that there is at least one person in the population of people without *G* (i.e. the referent strata) who would have the outcome directly due to *E*; similarly the arrow between *G* and *D* implies that there is at least one person in the population without *E* who would have the outcome due to *G*. The arrow connecting the interaction *E* × *G* implies that there is at least one person in the population for whom if both *E* and *G* are present, their outcome would be different to the situation in which they had either *E* or *G* alone. We do caution that although it is convenient to think of nodes as people, the DAG is actually graphically encoding relationships of conditional dependence and independence between variables.
- This conceptual depiction of interaction allows for the effect of E × G to be either positive or negative, accommodating positive or negative interaction. This is standard in causal diagrams, in which arrows do not depict whether causal effects are positive or negative.
- The interaction node *E* × *G* is also a mediator of the effect of *E* on *D* (and *G* on *D*). Although some may see this as confusing the concepts of interaction and mediation, we believe that this actually makes explicit that the total effect of *E* on *D* is split into a direct effect and an additional effect due to interaction with *G* (and similar with the effect of *G* on *D*).
- Because the DAG is non-parametric, this representation can be used to represent exposures and outcomes on any scale. The representation is thus also agnostic to the selected type of effect measure, including whether an absolute or relative measure is chosen, and the assumed model of interaction (additive or multiplicative). As stated by Hernán,^10^ the DAG is drawn based on an understanding of the biological phenomena. Thus, if an interaction is suspected on one scale or another, it can be denoted in the DAG using the interaction node.

As a specific example of Point (vi), consider the case in which the outcome D and exposures E and G are all binary. In this case the E × G node could represent an assumed difference in the causal risk difference for the effect of E on D, according to the value of G, i.e.

$$Pr\left(D^{e=1,g=0}=1\right)-Pr\left(D^{e=0,g=0}=1\right)\neq Pr\left(D^{e=1,g=1}=1\right)-Pr\left(D^{e=0,g=1}=1\right)$$

Or equivalently, the E × G node represents a difference in the causal risk difference for the effect of G on D, according to the value of E. This can be parameterized using a generalised linear model (GLM) with a binomial response distribution and natural logarithm link with corresponding model equation:

$$g\left(E\left(D_{i}\right)\right)=ln\left(\pi_{i}\right)=\beta_{0}+\beta_{1}E_{i}+\beta_{2}G_{i}+\beta_{3}{\left(E\times G\right)}_{i}$$

where $\pi_{i}$ represents the predicted probability that response D = 1 for individual i, conditional on their covariate pattern $E_{i},G_{i},{\left(E\;\times \;G\right)}_{i}$.

As a second example, consider the case in which the outcome D and exposures E and G are all continuous. In this case the E × G node may reflect the assumption that the predicted mean response, conditioned upon the value of either exposure variable, differs according to the value of the other exposure variable. This can be parameterized using a GLM with Gaussian response distribution and identity link with model equation:

$$g\left(E\left(D_{i}\right)\right)=\mu_{i}=\beta_{0}+\beta_{1}E_{i}+\beta_{2}G_{i}+\beta_{3}{\left(E\times G\right)}_{i}$$

In both examples above—i.e. regardless of the scale of the response and exposure variables—inclusion of the interaction node in the DAG simply represents an assumption that $\beta_{3}\;\neq \;0$, where $\beta_{3}$ represents the assumed interaction effect(s) (on the scale of interest). This generalizes naturally to other GLMs and combinations of exposure scales.

A potential limitation of this graphical depiction is that the interaction node initially appears to be completely determined by the two parent nodes, which appears to violate the principle that causal arrows comprise a combination of systematic and random effects, meaning all endogenous nodes are affected by both exogenous disturbances and endogenous ancestors of the node.

However, the implied relationship between each parent E,G and the descendant E × G is consistent with a causal relationship as defined probabilistically in DAG theory. For example, assuming binary exposures, conventional DAG theory defines causality of parent E upon E × G as:

$$Pr\left[{E\times G}^{e=1}=1\right]\neq Pr\left[{E\times G}^{e=0}=1\right]$$

and for parent G upon E × G as:

$$Pr\left[{E\times G}^{g=1}=1\right]\neq Pr\left[{E\times G}^{g=0}=1\right]$$

And both of these conditions are met by the included interaction node E × G.

Any potential collider stratification bias when conditioning on *G* × *E* is not problematic since E, G and E × G are the three exposure variables of interest in such a model and cause no confounding bias via influencing minimal adjustment sets. For example, when considering the causal effect corresponding to the arrow from G to D, assuming all known confounders of G and D have been controlled for, the presence of the E × G interaction node initially creates an open back-door path (since we are conditioning on the E × G collider); however, adjusting for *E* in this model closes the back-door path. It is possible that there are factors that influence the likelihood of both exposures occurring concurrently (marked by an arrow into the interaction *E* × *G* node) and also influence disease risk (marked by an arrow into the disease *D* node); such a factor would create a back-door path and this would be explicitly visualized in the DAG (see Figure 2c). Continuing with our example of smoking (*E*), asbestos (*G*) and lung cancer (*D*), a potential confounder (*C*) would be a factor that increases the likelihood of both smoking and asbestosis exposure, such as socio-economic status. Although this back-door path could also be captured by arrows from *C* to both *E* and *G* if the *E* × *G* node were omitted, the interaction node prompts the researcher to think about factors that affect both exposures simultaneously.

## Effect modification

Effect modification occurs when there is interest in the causal effect of a single exposure on an outcome and this causal effect depends on the value of a third variable, termed an effect modifier. The key distinction between interaction and effect modification is that with effect modification, interest is in the causal effect of only a single exposure, whereas with interaction, interest is in both causal effects. This definition of effect modification corresponds to what was previously termed ‘exposure modification’.^11^

VanderWeele and Robins^1^ depict effect modification as shown Figure 1, which was also used to depict interaction. In that example, the exposure E was a medication, the outcome D was hypertension and the variable G was a genotype that modifies the effect of E on D. As an example, G may be a polymorphism in a gene encoding a cytochrome P450 enzyme, with the polymorphism causing the drug to be metabolized either more or less quickly than usual, decreasing or increasing, respectively, the causal effect of E on D. There is nothing in this DAG that suggests that the effect of genotype (*G*) on disease (*D*) is present only if the medication (*E*) is taken.

**Figure 3:**
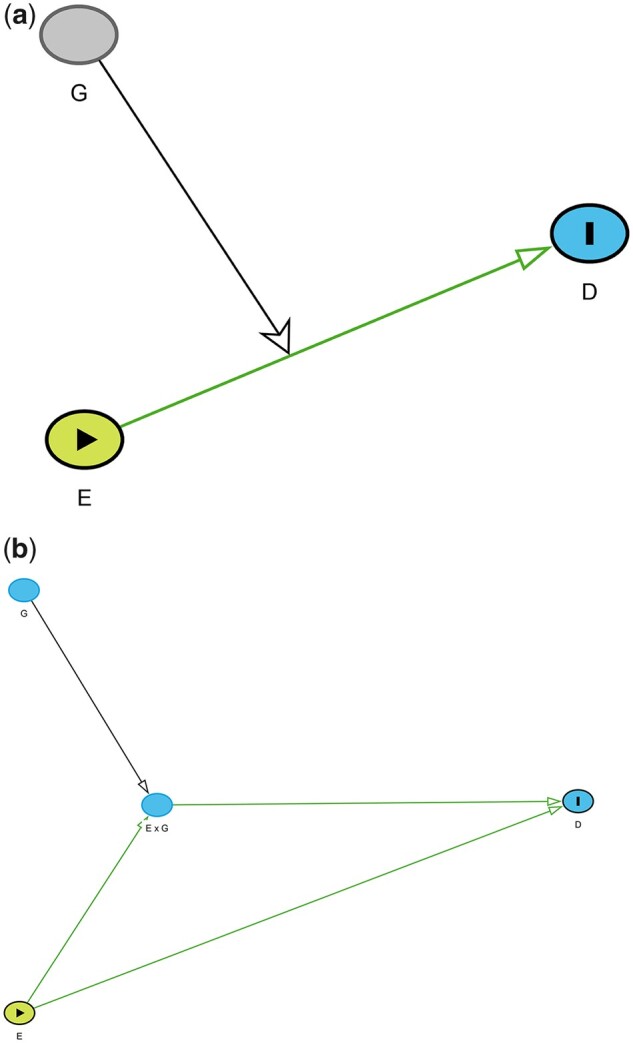
(a) Proposal by Weinberg to capture effect modification by genotype (*G*) of medication (*E*) on hypertension (*D*). (b) DAGs representing the independent effect of exposure *E* on outcome D and modification of this effect by *G*.

Weinberg^4^ suggested that such effect modification could be captured by the DAG in Figure 3a. However, as mentioned before, this representation can be neither depicted using available DAG software nor interpreted using standard DAG theory.

We propose that effect modification could be graphically depicted as shown in Figure 3b. Here, inclusion of the E × G node represents an assumption that the causal effect of E on D is modified by G.

We are not aware of this graphical representation being previously suggested. This representation shares several key features of the proposed representation of interaction and also has some differences:

- The assumed effect modification can be graphically depicted and interpreted using existing software and theory. Because interest is in the causal effect of exposure *E*, assuming effect modification by *G*, causal diagrams can be constructed by defining both E and E × G as exposure variables. This allows for the specification of additional, biasing paths involving other causally related variables, and the identification of conditional independencies and minimum adjustment sets.
- It is possible to specify either that the effect modifier G has a direct causal effect on outcome D, or that it does not, depending on the assumed causal model. Because G is not an exposure variable in the effect modifier case, its causal relationship with D is relevant only for identifying adjustment sets. In either case - i.e., whether *G* does or does not have a causal effect on *D* — the minimum sufficient adjustment set for estimating the total effect of E and E × G on *D* contains *G*, as necessary to estimate causal effects of E and E × G conditioned upon *G*. Given that a DAG should code all relevant causal effects in order to accurately specify all back-door paths and hence the minimal adjustment set, we propose that the term ‘effect modification’ should only be reserved for situations in which the second factor does not have a direct effect on the outcome, i.e. Figure 3b. If the second factor does have a direct effect on the outcome, then the term ‘interaction’ should be used; this would provide some clarity on terminology.
- If the genetic polymorphism *G* only affects disease *D* in the presence of the medication *E*, then exchangeability on *G* is not required and it could be argued that *G* should not appear in the DAG at all. However, we would argue that including *G* in the DAG provides clarity that the *E* × *G* node is not simply a mediator of the effect of *E* on *D*, and also prompts the researcher to consider potential back-door paths that might influence both *G* and *D*, e.g. ethnicity might influence the frequency of the polymorphisms as well as disease risk.
- The DAG depicts the assumption that *E* has a direct causal effect on *D*. With the *E* × *G* node included, this ‘main’ effect represents the average causal effect of *E* for a subpopulation with specified (e.g., reference) values of *E* and *G*, rather than representing the average causal effect of *E* in the population. Conditional on *E*, the *E* × *G* node then represents the average causal effect for one or more other, joint values of *E* and *G*.
- This depiction allows for the direction of effect modification to be positive or negative, for exposures and outcomes to be on any scale, and for different assumed models of effect modification.

**Figure 4:**
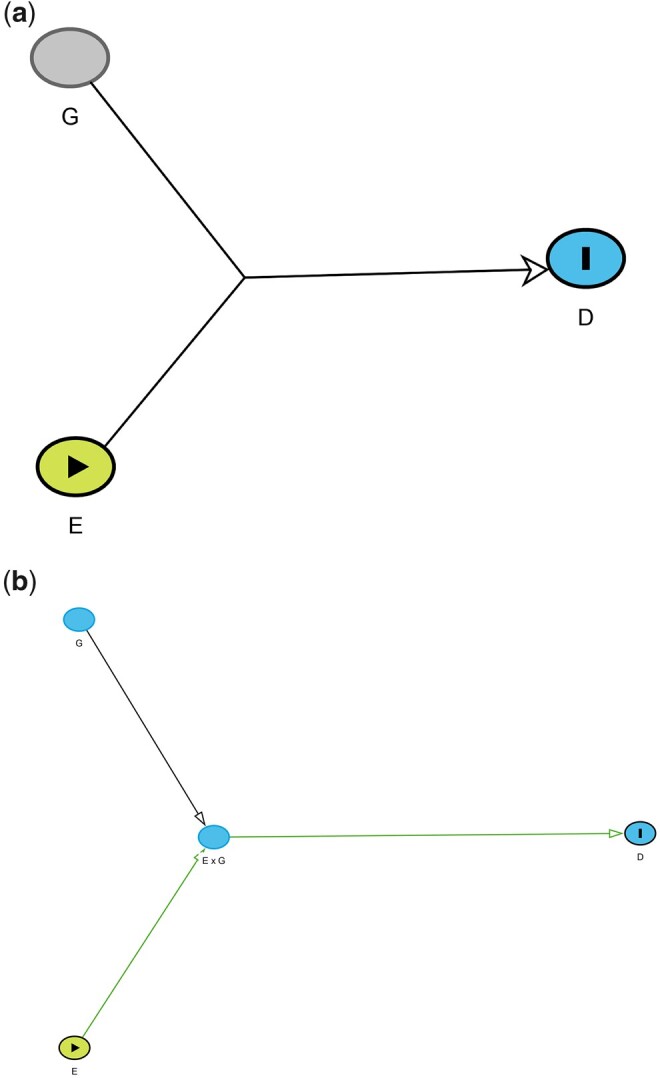
(a) ‘Pure’ interaction in which neither *G* nor *E* has a direct effect on *D* and they only have an effect when present together. (b) ‘Pure’ interaction in which the causal effect of exposures E and G on outcome D is only present for specific joint values of E and G.

Weinberg^4^ proposed a further diagram to capture ‘pure’ interaction in which neither G nor E has a direct effect on D and they only have an effect when present together (see Figure 4a).

We suggest that this ‘pure’ interaction could be captured by the DAG shown in Figure 4b. An example of this is phenylketonuria in which phenylalanine in the diet (E) and the absence of the enzyme to break it down (phenylalanine hydroxylase, G) are both needed in order for a disease to manifest (intellectual disability, D).

Specific features of this representation include:

- The DAG depicts the assumption that neither *E* nor *G* has a direct effect on *D*. Because interest is only in the causal effect of a specified combination of values of exposures *E* and *G*, causal diagrams can be constructed by defining only the node *E* × *G*, as an exposure variable. However, we argue that drawing the DAG including both the *E* and *G* nodes in this way is also helpful for clarity about the causal effects.
- Using conventional DAG theory, this representation defines a minimum sufficient adjustment set for estimating the total effect of *E* × *G* on *D* that contains both *E* and *G*, as necessary to estimate the causal effect of *E* × *G* conditioned upon *E* and *G*. However, as is usual, when interpreting regression parameter estimates, interest is restricted to the single exposure variable *E* × *G*, representing the ‘pure’ interaction effect.
- The DAG depicts the assumption that *E* and *G* only have a causal effect on *D*, conditional on the values of *E* and *G*, i.e. is a causal effect only for selected, joint values of *E* and *G*.

DAGs have been a tremendous boon in clarifying thinking around causal inference and articulating the fact that epidemiologists are indeed interested in biological cause and effect relationships, not just abstract ‘association’. It has been a gap that there is no easy way to capture biologically meaningful interaction or effect modification using conventional DAG models. Our proposal is hopefully a step forward in this respect.

## Author contributions

J.A. prepared the draft manuscript and is guarantor for this work. E.H. and C.O. contributed to the mathematical and statistical methods.
