# Correction to: A proposal for capturing interaction and effect modification using DAGs

**DOI:** 10.1093/ije/dyac153

**Published:** 2022-07-20

**Authors:** John Attia, Elizabeth Holliday, Christopher Oldmeadow

First published online 13 June 2022, *International Journal of Epidemiology*, https://doi.org/10.1093/ije/dyac126

The sentence “For example, when considering the causal effect corresponding to the arrow from *G* to *D*, assuming all known confounders of *G* and *D* have been controlled for, the presence of the *E × G* interaction node initially creates an open back-door path (since we are conditioning on the *E x G* collider); however, adjusting for *G* in this model closes the back-door path.” Should have concluded “however, adjusting for *E* in this model closes the back-door path”

Further, the paragraph “We propose that effect modification could be graphically depicted as shown in Figure 3b. Here, inclusion of the *E × G* node represents an assumption that the causal effect of *E* on *D* is modified by *G*.” should have appeared in the discussion of Figure 3, earlier in the paper.

The paper has been corrected.

